# A review of Constraint-Induced Therapy applied to aphasia rehabilitation in stroke patients

**DOI:** 10.1590/S1980-57642009DN30400003

**Published:** 2009

**Authors:** Joana Bisol Balardin, Eliane Correa Miotto

**Affiliations:** 1 MD, Post-Graduate Program in Neurology, Department of Neurology, the University of São Paulo Medical School, São Paulo SP, Brazil.; 2 PhD, Department of Neurology and Division of Psychology, University of São Paulo Medical School, São Paulo SP, Brazil.

**Keywords:** aphasia, stroke, rehabilitation of speech and language disorders

## Abstract

Constraint-induced aphasia therapy (CIAT) is an intensive therapy model based on the forced use of verbal oral language as the sole channel of communication, while any alternative communication mode such as writing, gesturing or pointing are prevented.

**Objectives:**

This critical review involved the analysis of studies examining CIAT applied to stroke patients.

**Methods and Results:**

Using keywords, the Medline database was searched for relevant studies published between 2001 and 2008 (Medline 2001-2008). The critical evaluation of the articles was based on the classifications described by the ASNS (Cicerone adaptation). Two studies were categorized as level Ia, two as level II and one study as level IV.

**Conclusions:**

These recommendations should be interpreted with caution, given the small number of studies involved, but serve as a guideline for future studies in aphasia therapy.

## Abstract (pt)

A Terapia Induzida por Contenção aplicada à reabilitação da afasia é um modelo terapêutico intensivo que presume o treinamento da linguagem oral como exclusivo canal de comunicação verbal, enquanto métodos alternativos como a escrita, o desenho e os gestos não são permitidos.

**Objetivos:**

Revisar sistematicamente os estudos que examinaram a aplicabilidade da Terapia Induzida por Contenção em pacientes afásicos pós AVC.

**Métodos e Resultados:**

Os estudos relacionados ao tema foram selecionados na base de dados Medline, por meio de descritores, entre os anos de 2001 e 2008. A avaliação crítica dos artigos foi realizada de acordo com a classificação descrita pela ASNS (adaptado por Cicerone). Dois estudos foram categorizados como Ia, dois estudos receberam classificação II e um estudo foi categorizado no nível IV.

**Conclusões:**

Dado o limitado número de estudos existente, as recomendações sugeridas devem ser interpretadas com precaução, porém fornecem importantes diretrizes para futuros estudos sobre a terapia de afasia.

Aphasia is defined as the loss of the ability to produce or comprehend language due to focal brain damage to the language-dominant cerebral hemisphere, and constitutes a frequent sequelae of left hemispheric stroke.^1^ In the acute phase, nearly two fifths of stroke patients suffer from aphasia,^2^ and approximately 40% to 60% of these progress to the chronic stage.^3^ Clinically, aphasia is characterized by paraphasias, word finding difficulties, different levels of impaired comprehension, writing and reading problems.^4^ Some degree of dysarthria can also co-exist with aphasia, especially when observed in actual practice.^5^ These impairments impact the quality of life of stroke survivors, including their capacity to maintain reciprocal relationships with others, hampering work productively and participation in important life events.^6^

The impact of aphasia on the lives of stroke survivors has led to a number of studies in different areas of research, designed to test the effects of behavioral interventions. There is a general consensus that aphasia therapy is helpful in improving specific measures of language function and communication in a variety of settings. Most of the direct treatments rely on the principle that the more often a patient produces a particular correct response, the more often that person will be able to produce that same correct response independently in the future.^7^ Although speech therapy is widely accepted as an effective means of managing language problems in stroke survivors, some issues remain controversial in aphasia therapy literature. Such issues include the lack of generalization of treatment effects to improvements of functional communication in everyday life, and the extent to which improvements occur after the 6-month spontaneous recovery phase.^8^

Recently, a new approach to investigate language reorganization in aphasia was proposed which employs therapy techniques that promote improved functions in a matter of days or weeks, even in chronic stroke patients, within a pragmatic therapeutic environment. Drawing on basic research in behavior neuroscience, constraint-induced aphasia therapy (CIAT) is based and modeled on a physical rehabilitation program for recovery of motor deficits called constraint-induced motor therapy (CIMT).^9^ CIMT is based on the notion that the potential rehabilitation of the affected limb is detrimentally influenced by the compensatory use of the unaffected limb, through a process of learned non-use.^10^ Thus, the principle of CIMT is to prevent extremity disuse by forcing patients to utilize the affected muscles in a massed practice routine, while avoiding compensatory non-use techniques.^11^ Even in patients with chronic stroke, CIMT has led to clinical improvements associated with cortical plasticity observed on fMRI.^12^ In aphasic patients, non-vocal communication channels (e.g. gesturing, drawing or writing) have been considered compensatory mechanisms that induce a form of verbal learned non-use, suggesting the potential value of the transfer of the CIMT approach to support language recovery in stroke.^13^ Although the exact neurobiological principles underlying the positive effect of CIMT are unknown, evidence from animal and human research have shown that forced use of affected limbs/functions can promote cortical reorganization though processes such as strengthening of the remaining neuronal connections within damaged cell assemblies and unmasking silent neural pathways.^9^

## Principles of CIAT

CIAT was first proposed by Pulvermüller et al.^14^ as a therapeutic approach that included the principles of massed practice (3 to 4 hours per day for 10 consecutive days), shaping (the difficulty of the required verbal actions is gradually increased according to the patients’ needs) and constraint of compensatory (nonverbal) communication strategies.

The principal technique entailed a therapeutic language exercise, closely related to everyday communication, in the form of a game of cards bearing drawings of objects, played by 2-3 patients and a therapist.^15^ In sum, 4-5 cards with pictures of different exemplars of a semantic category are given to each participant. The goal of the task is to collect as many pairs of matching cards as possible. For each “turn” one participant (the speaker) ask the other participant (the receiver) if he/she has a particular card, and the receiver answers with an explicit reply.^16^

The shaping technique is introduced gradually, according to the evolution of each patient. In the initial phase, all approximately relevant utterances are acceptable. Subsequently, the therapist can specify the use of the names of the co-players or the addition of politeness utterances. For advanced patients, syntactic sentence frames were required instead of 1- or 2-word utterances.^14^ Therapists can provide as much cuing as necessary to yield a successful turn.^16^

The constraint technique was defined as a method of limiting the patient’s response to spoken verbal production only. This sometimes includes screens between players, to prevent them from seeing each other’s cards or communicating using gestures. Use of alternative modes of communication is forbidden (e.g., writing, gesturing, pointing, etc.).^14,17^

In the first publication,^14^ the authors provided evidence from a controlled randomized trial for the effectiveness of CIAT. They used an experimental group of 10 inpatients with chronic aphasia treated for 3 hours per day for 10 consecutive days, and a control group for comparison containing 7 chronic inpatients who received “conventional” speech and language therapy for the same total number of hours as the experimental group, but instead distributed over 3 to 5 weeks. CIAT was shown to result in improved performance over the standardized language test and in terms of the quality of everyday communication assessed by clinicians blinded to group status. The comparison group demonstrated no improvements in any of these measures.

Since its first publication, the CIAT protocol has been replicated and modified by studies adopting different scientific methodologies. The purpose of this article was to review relevant research regarding the effects of CIAT on the management of language impairment in stroke patients. The authors sought to contribute to the urgent need of using evidence-based practice in aphasia therapy, to better evaluate the quality of the study results and their power of replicability in clinical use. Based on standards published by the American Association of Neurologic Surgeons – AANS^18^ for classification of research studies, adapted by Cicerone et al.^19^ to evaluate evidence from cognitive rehabilitation programs, we sought to classify the level of evidence and to produce recommendations for interventions in language rehabilitation.

## Methods

In the current review, the MEDLINE (2001-2008) database was searched using a combination of the following terms: aphasia therapy, intensive language therapy, constraint induced aphasia therapy, neurorehabilitation, and language recovery. The year 2001 was selected as a cutoff year because the first adaptation of constraint induced movement therapy for language therapy in aphasia dated from 2001. Research articles investigating any aphasia type were included because limiting the review to a specific aphasia type was considered premature, yielding too few articles to compile a meaningful review. In addition, controlling for severity was not possible because of the diverse testing protocols employed by the studies. The search was restricted to English language reports describing clinical trials of language rehabilitation in aphasic patients. We also conducted a manual search of the references listed in the resulting articles to identify other appropriate articles. Review articles were excluded from this review.

The critical evaluation of the articles for level of evidence was based on the classifications described by ANNS^18^ and adopted by Cicerone et al.^19^ These levels (I-IV) are organized as a hierarchy that represents the confidence generated by the study results. Class I comprises well-designed, prospective, randomized controlled trials. Class Ia includes well-designed, prospective quasi-randomized assignment to treatment conditions (e.g., alternating conditions). Class II incorporates: prospective, nonrandomized cohort studies; retrospective, nonrandomized case-control studies; clinical series with well-designed controls that permitted between-subject comparisons of treatment conditions; all other controlled studies in a representative population. Class III includes clinical series without concurrent controls and studies reporting one or more case study that used appropriate single-subject methods. Class IV comprises evidence from uncontrolled studies, case series, case reports, or expert opinion. Final acceptance of evidence classification was based on total agreement between the 2 reviewers. After review of the article and classification of level of evidence, reviewers then provided recommendations based on the strength of the levels of evidence found in the study on the feasibility of constraint induced therapy models for language rehabilitation in aphasia. The recommendations were classified, according to Ciccerone et al.,^19^ as either (1) practice standards, (2) practice guidelines, or (3) practice options, based on the body of evidence available.

### Review of the literature

A total of 16 studies were initially found. Two articles were descriptive reports and were therefore excluded.^13,20^ Four articles reported experimental or quasi-experimental studies,^14,16,17,21^ and one study reported multiple cases that received the same CIAT treatment.^22^ The remaining nine studies reported single-case or case series studies on the cortical network associated with the effects of CIAT on aphasic stroke patients.^23-31^ Each article was independently reviewed and the methodological characteristics of the 5 effectiveness studies were described,^14,16,17,21,22^ along with the classification of each, according to levels of evidence.

### Clinical data and levels of evidence

Details of the 5 primary studies are summarized in Table 1. Two studies were categorized as level Ia;^14,21^ two studies as level II^16,17^ and one as level IV^22^. The studies categorized as level II included two groups, one containing controls that received traditional intervention. Age, gender ratio and years of education were similar across all studies selected. Large differences in time since left hemisphere stroke were evident, but patients of all studies were at a chronic stage (6-12 months post onset). Classification or description of the stroke (localization, number of episodes, hemorrhagic or ischemic) was not consistently reported across all the studies. Right hemiplegia or hemiparesis and a general cognitive assessment were only reported in one study,^19^ while all of them report the exclusion of patients with additional neurological and psychiatric diseases or cognitive and perceptual deficits that prevented them from fully participating in aphasia testing or in therapeutic training procedures. Aphasia syndrome and severity were heterogeneous, although in general, patients had non-fluent moderate language disorders. Comorbid apraxia of speech was evaluated in 2 studies.^16,21^

**Table 1:** Reviewed studies.

Study Study design Level of evidence	n	Experimental condition (EC)	Control condition (CC)	Outcome measure/s	Results	Follow-up results
Pulvermüller et al. (2001)^14^	EC n=10, CC n=7A=55.4, 53.9G=60%, 85%E=11.1, 10.9AT=heterogeneous, except global aphasiaAS=heterogeneous (mild to severe)TO=98.2, 24.0	CIAT in the context of a therapeutic game activity for 10 days, 3 to 4 hours per day (23 to 33 hours of therapy; mean, 31.5) performed in groups of 2 or 3 patients plus the therapist.	Conventional aphasia therapy involving naming, repetition, sentence completion, following instructions, and conversations on topics of the patients' own choosing, administered for 3 to 5 weeks (20 to 54 hours; mean, 33.9).	AAB Token Test Comprehension Repetition Naming CAL	Between-group differences were observed on AAB (p<0.04), and CAL (p<0.01). Significant improvements in the group that received CI aphasia therapy p<0.001) were observed in 4 of 5 tests (exception was repetition), whereas the group that received conventional aphasia treatment did not reveal significant overall improvement.	No follow-up data.
Randomized controlled trial						
Level Ia						
Meinzer et al. (2005)^17^	EC n=12, CC n=15A=50.1, 52.1G=66%, 53%E=not availableAT=heterogeneous, except global aphasiaAS=heterogeneous (mild to severe)TO=46.2, 47.9	CIATplus that includes additional exercises not present in the original protocol, to be performed at home, and the use of photographs instead of pictures as objects in the game. Therapy was administered for 30 hours over a 2-week period (3 hours/day).	CIAT original.	AAB Token Test Comprehension Repetition Naming	Within-group improvements were observed for both groups across all AAB tests, CAL and CETI (all ps<0.0001). Between-group differences were not observed.	In both groups, improvements remained stable throughout the 6-month follow-up period. Improvements in CAL and CETI rated by relatives were more marked for patients in the CIATplus group (p<0.01).
Non-randomized controlled trial				CAL		
Level II			Therapy was administered for 30 hours over a 2-week period (3 hours/day).	CETI		
Maher et al. (2006)^16^	EC n=4, CC n=5A=48, 58G=50%, 60%E=15, 15AT=heterogeneous with significant word retrieval deficitsAS=heterogeneous (mild do severe)TO=38, 35	CILT with the introduction of a physical constraint (a visual barrier on the table between the participants so they could not see each other except for eye contact) to force the use of solely spoken communication. Additional levels of task difficulty based on the nature of required response was developed.	Conventional aphasia therapy (PACE), in the same context of game activity used in CILT. Therapy was administered for 24 hours over two-weeks (3 hours/day, 4 days/week).	WAB Aphasia Quotient BNTAction Naming Test Apraxia Battery forAdults-2Narrative discourse sample (Cinderella retelling)	Between-group differences were not found. Within-group improvements were observed for both groups on the WAB (p=0.004), BNT (p=0.006) and Action Naming Test (p=0.056). Qualitative analysis of the discourse showed that CILT participants exhibited more consistent improvements than patients under control conditions.	In both groups, improvements remained stable throughout the 1-month follow-up period (p>0.05).
Non-randomized controlled trial						
Level II		Therapy was administered for 24 hours over two-weeks (3 hours/day, 4 days/week).				
Meinzer et al. (2007b)^21^	EC n=10, CC n=10A=50.2, 62G=70%, 90%E=11, 11AT=heterogeneousAS=heterogeneous (mild to severe)TO= 30.7, 46.5	CIATplus (Meinzer et al., 2005) applied by trained psychologists. Therapy was administered 3 hours/day for 10 consecutive days.	CIATplus applied by trained laypersons. Therapy was administered 3 hours/day for 10 consecutive days.	AABToken TestComprehensionRepetitionNamingWriting	Within-group improvements were observed for both groups across all AAB subtests (all ps<0.05). Between-group differences were not observed.	No follow-up data.
Randomized controlled trial						
Level I						
Szaflarski et al. (2008)^22^	Sb=P1, P2, P3A=range 58-64G=range 12-24 E= 14.6AT=P1: non-fluent aphasiaP2: fluent aphasia with moderate comprehension impairmentP3: non-fluent aphasiaAS=moderate to severe	CIAT (Pulvermüller et al., 1991) with a hierarchy of individual skill levels for semantic, syntactic, and phonological language production. Treatment was administered 3 hours/day for 5 days.	No control group included.	BDAE-3 BNT+ Grammatical Word Discrimination Commands Complex Ideational Material	Substantial improvements in comprehension and verbal skills were noted in 2 patients (total number of words and in number of utterances for story-retell task), and no subjective improvements on mini-CAL were noted by any of the participants.	No follow-up data.
Multiple single- subject design						
Level IV				Narrative story retell Mini-CAL		

Age, A (mean years); Gender, G (% male); E, education (mean years); AT, aphasia type; AS, aphasia severity TO, time post onset (mean months); CIAT, Constraint Induced Aphasia Therapy; CILT, Constraint Induced Language Therapy; EC, experimental condition; CC, control condition; SG, single group; Sb, subjects AAB, Aachen Aphasia Battery; BNT, Boston Naming Test; CAL, Communicative Activity Log; CETI, Communicative Effectiveness Index; WAB, Western Aphasia Battery; BDAE-3, Boston Diagnostic Aphasia Exam-3.

### Study design, interventions and outcome measures

Two studies were randomized controlled trials,^14,21^ two non-randomized controlled trials,^16,17^ and one a case series report.^22^ Whenever applicable, groups were reasonably balanced in terms of aphasia severity (Table 1). None of the studies described details about the randomization procedure employed. Three studies^14,16,21^ used a blinded assessor to determine the outcome measures. Two studies comprised an experimental group (CIAT), and a control group that received a conventional intervention.^14,16^ One study compared the original CIAT protocol with a modified version^16^ whereas the other study investigated the CIAT protocol applied by trained psychologists, comparing this to trained laypersons.^21^ Absence of differences between groups at baseline was reported in two studies.^14,16^ Follow-up assessment was performed in two studies,^16,17^ but was only analyzed in one.^17^ Both the experimental (CIAT) and control intervention were balanced for duration (hours of therapy) and frequency (sessions per week) in three studies.^16-18^ Principal outcomes were measures of communication, including oral expressive language, oral receptive language and functional communication. Pre and post-treatment measures included standardized language tests in all studies. Two studies incorporated linguistic analysis of narrative discourse^16,22^ and three studies included a measure of progress in everyday communication.^14,17,22^

### Findings

Overall, the studies reported improvements in language functions for both groups: the experimental (CIAT) and control (conventional aphasia therapy or a modified version of CIAT) groups. One study^14^ found that only the experimental group showed significant improvements in almost all language tests and in communicative daily situations. The only study that followed-up subjects 6 months after the training period revealed that patients in the experimental group exhibited greater stability of treatment gains than the control group.^17^ More consistent improvements in narrative discourse measures were also observed for the CIAT group compared to controls.^16^

### Grades of recommendation

There are no specific recommendations to be made based on the available evidence for studies in this category. The class Ia studies^14,21^ appeared promising but lacked the detail needed to identify which active ingredients may have resulted in the improvements seen. Further studies of CIAT methodology may prove useful for cognitive recovery in aphasics.

## Discussion

The two randomized controlled trials included in the systematic review^11,18^ were ranked level Ia, using Cicerone’ rules of evidence. The remaining 11 studies were ranked level IV due to decreased rigor of the research designs.

Previous systematic reviews concerning the efficacy of formal speech and language therapy for stroke patients had encountered difficulties in examining studies as a group. Greener et al.^31^ examined 12 trials and found most of them to be relatively old with poor or inaccessible methodological quality. None of the trials were detailed enough to allow complete description and analysis, and none of the results were able to determine whether or not formal speech and language therapy was any more or less effective than informal support in aphasia therapy. The authors concluded that decisions about the management of patients must therefore be based on other forms of evidence.

Many methodological issues seem to permeate the evaluation of clinical trials in aphasia rehabilitation. One of them includes difficulties concerning randomization. This is because it is well established that people with communication disorders are a heterogeneous group,^32^ and therefore assignment to different group treatments could lead to a high risk of having different aphasia syndromes, severities and comorbidities (e.g. apraxia of speech) in the two groups. Another aspect is related to the prediction of a specific outcome. Communication is such a complex, high order behavior that even when the cause is known, such as in stroke aphasia, it is hard to determine the impact of intervention on patients’ language use.

Considerations from the two previously published reviews^13,20^ on the adaptation of CIMT to cognitive functions, especially language, are also relevant. The authors emphasized the restorative role of CIAT and its neurobiological basis supported by studies of brain function. They reported that reorganizational changes investigated through magnetoencephalography (MEG)^23^ and electroencephalography (EEG)^24^ have been found in patients who significantly improved language function with CIAT. Although we did not extensively review the other seven single-case or case series studies^25-30^ on the cortical network associated with the effects of CIAT (they were beyond the scope of our primary aim to review only effectiveness studies), their general results reinforce the finding of brain enhanced activity associated with treatment progression.

Most studies conducted to date have focused on the remediation of language in general, which is necessary and appropriate since aphasia impacts all aspects of language processing. The one practice guideline resulting from this review was in this domain. This guideline was the result of a study by Pulvermüller et al.^14^ However, their study was unable to answer the question of how much each feature of the constraint-induced therapeutic approach to language disorders contributed to the success achieved. Clearly, this new therapeutic approach is based on three related principles, each of which could be a sufficient rather than necessary condition for therapeutic success. These different principles include massed practice, shaping and constraint of compensatory (nonverbal) communication strategies. On the basis of the present data, we cannot rule out the possibility that, for example, conventional therapy performed in a massed-practice fashion could also result in marked behavioral improvement within a few days. The 3 principles’ individual influences should be quantified in future investigations.

Beyond the analysis of levels of evidence, a summary of treatment results are provided in Table 1. In the first study,^14^ overall improvement was significant for the CIAT treatment group; 3 of 4 subtests of the Aachen Aphasia Battery (AAB) showed significant improvement. The group that received the standard treatment improved on one of the subtests, but no overall change occurred. The amount of life situation communication as measured by Communicative Activity Log (CAL) ratings of patients and therapists was significantly improved in the CIAT treatment group but not in the control group. This language improvement reported within a two-week treatment period is remarkable, but limitations include the small patient sample and the absence of information about long-term retention of the benefits. In the study of Meinzer et al. (2005),^17^ comparing standard CIAT and a modified version called CIAT Plus, scores on AAB and CAL improved significantly in both intervention groups immediately after the training. Improvement in language tests was not correlated with age or duration of aphasia. In the 6-month follow-up assessment, no decline in retention of AAT scores was evident, but no further improvement in language function was found in either group. The authors suggested that perhaps the subjects had reached the maximum function they were capable of with this type of training by the end of the treatment period. In the study by Maher et al. (2006),^16^ whereas both interventions yielded positive outcomes, CIAT participants showed more consistent improvement on Western Aphasia Battery (WAB) measures and clinician judgments of narrative discourse. As the only difference between the two groups was the availability of alternative methods to support communication in the control group, the extent of the CIAT advantage over the control therapy remained unclear. The other study by Meinzer et al. (2007b)^21^ showed that although between-group differences were not found, aphasic patients trained by experienced therapists as well as those trained by laypersons presented improvements on AAB subtests. These results are extremely important in revealing the feasibility of using CIAT protocols in public services, since intensive practice schedules are inaccessible to many potential clients. In their latest study,^22^ the authors demonstrated that a CIAT program based on individual skill levels for semantic, syntactic, and phonological language production improved BDAE-3 test measures in a case series of three patients.

Based on these data it would be premature to conclude that there is a clear advantage of applying constraint principles to aphasia rehabilitation over other types of intensive intervention. However, the data suggest that some aspect of the CILT approach confers additional benefit. Whereas intensity has been reported to be an important factor in the outcomes of aphasia rehabilitation,^33^ the study by Maher et al.^16^ provided significant evidence that intensity alone cannot explain the positive differences between the two groups’ performance, because intensity was controlled. Another important finding of this study was that the continued impact of CILT after therapy proved short-lived (e.g. 3 months follow-up period). This is consistent with findings reported elsewhere^16^ and in the motor literature.^34^
